# Clinical efficacy of tirofiban combined with a Solitaire stent in treating acute ischemic stroke

**DOI:** 10.1590/1414-431X20198396

**Published:** 2019-09-16

**Authors:** Yang Zhang, Qing-Qing Zhang, Cong Fu, Lei Wang, Guan-Qun Zhang, Pei-Wei Cao, Guo-Fang Chen, Xin-Min Fu

**Affiliations:** 1 Department of Neurology, Xuzhou Central Hospital, Xuzhou Hospital Affiliated to Medical College, Southeast University, Xuzhou, China; 2 Graduate School of Bengbu Medical College, Bengbu, China; 3 Department of Rehabilitative Medicine, Xuzhou Central Hospital, Xuzhou Hospital Affiliated to Medical College, Southeast University, Xuzhou, China

**Keywords:** Acute ischemic stroke, Solitaire AB stent, Endovascular thrombectomy, Tirofiban

## Abstract

This study explores the safety and effect of acute cerebral infarction treatment by microcatheter injection of tirofiban combined with a Solitaire AB stent and/or stent implantation. Emergency cerebral angiograms showing the responsible vascular occlusion of 120 acute cerebral infarction patients who underwent emergency endovascular thrombectomy were included in the study. These patients were randomly divided into two groups using the random number table method: treatment group (n=60) that received thrombectomy (with cerebral artery stents) combined with intracerebral injection of tirofiban and control group (n=60) that only received thrombectomy (with cerebral artery stents alone). The baseline data, cerebral angiography before and after surgery, hospitalization, and follow-up results of patients in these two groups were compared. Furthermore, the incidence of major adverse cerebrovascular events of these two groups was compared (90-day modified Rankin scale, a score of 0–2 indicates a good prognosis). The difference between baseline clinical data and brain angiography between these two groups was not statistically significant. Patients in the treatment group had a higher prevalence of thrombolysis in cerebral infarction grade 2b/3 than patients in the control group (88.3% (53/60) *vs* 66.7% (40/60), P=0.036). Moreover, the National Institutes of Health Stroke Scale scores 7 days after surgery and the 90-day prognosis were all better for the patients who received tirofiban (P=0.048 and P=0.024). Mechanical thrombectomy with Solitaire AB stents in combination with the injection of tirofiban through a microcatheter appears to be safe and effective for the endovascular treatment of acute ischemic stroke.

## Introduction

Acute cerebral infarction is a common disease with high morbidity and mortality. Early thrombolytic therapy is one of the most effective methods to reduce the mortality and disability of acute cerebral infarction. (1) The purpose of thrombolysis is to open the occluded vessels in time and save the ischemic penumbra neurons (2), and this has been generally considered to be the best time for intravenous thrombolysis (within 4.5 h after the onset of acute cerebral infarction) (1). However, the efficiency of the revascularization (3) is approximately 30%, good prognosis is approximately 15%, and the time window is 4.5 h. Clinically, the majority of patients exceed the intravenous thrombolysis time window. Hence, investigators have attempted to perform arterial thrombolysis and endovascular thrombectomy for ischemic stroke at 8–12 h after the patient was last known to be well plus standard medical therapy (3). With the arterial injection of low dose tirofiban by a microcatheter, the patient had less bleeding risk and achieved a better clinical result, and this was combined with endovascular thrombectomy, when necessary, using a brain artery stent. Stent implantation was performed to open the occlusion of the cerebral arteries, and cerebral blood supply was resumed as soon as possible to reduce the risk of cerebral infarction, and the sequela of cerebral infarction, or death. In the treatment of atherosclerotic cerebral infarction, vascular endothelial injury and platelet activation can easily occur, which leads to the formation of *in situ* thrombosis and the reocclusion of blood vessels. The incidence of intracranial atherosclerosis in the Asian population is high (4,5).

In cases resistant to mechanical thrombectomy, several techniques have been introduced such as a rescue method including the use of other stent retrievers, intraarterial thrombolysis, stent implantation (6), balloon angioplasty (7), and double solitaire mechanical thrombectomy (8). Here, we present our experience of another rescue technique in which adjunctive tirofiban was injected through a temporarily deployed microcatheter after failure of initial mechanical thrombectomy. We evaluated the feasibility, safety, and angiographic and clinical results of this technique.

In recent years, the Solitaire AB stent mechanical thrombectomy combined with the injection of low dose tirofiban by a microcatheter in our hospital for the treatment of acute cerebral infarction has achieved good effects. The details of the report are presented, as follows.

## Material and Methods

### Study subjects

A total of 120 patients with acute cerebral infarction who were selected for complete thrombectomy of the blood vessel after emergency cerebral angiography from March 2012 to April 2016, were selected as the study subjects. Inclusion criteria were: patients 18–86 years old; clinical diagnosis of acute cerebral infarction and a National Institutes of Health Stroke Scale (NIHSS) of >3, which lasted for more than 30 min; an onset time of eight hours (posterior circulation not more than 12 h according to the situation); computed tomography (CT) or magnetic resonance imaging (MRI) that excluded cerebral hemorrhage or other obvious intracranial diseases; image-confirmed (CTA/MRA/DSA) intracranial artery occlusion (internal carotid artery, M1/M2 segment of the cerebral artery, anterior cerebral artery A1/A2, basilar artery, vertebral artery V4 segment, and posterior cerebral artery P1 segment); patients or family members who provided a signed informed consent. Exclusion criteria were: an NIHSS score of <3 or a NIHSS score that had significantly improved; a modified Rankin scale (mRS) score of >2 before the stroke; life expectancy of <3 months or unable to complete the study for other reasons; use of anticoagulants or antiplatelet drugs for a long time; digital subtraction angiography (DSA) inspection taboo (e.g.: serious contrast agent allergy or severe impairment of liver and kidney function or uncontrolled hyperthyroidism and diabetes); female patients who were pregnant or lactating; unable to complete the study due to mental illness; uncontrolled hypertension: pre-treatment systolic blood pressure >220 mmHg or diastolic pressure >110 mmHg; history of hemorrhagic cerebrovascular disease or bleeding tendency; important organ dysfunction or failure; patients or family members who did not provide a signed informed consent.

Among the 120 patients with acute ischemic stroke, 59 patients were male and 61 patients were female, and their age ranged within 27–86 years, with an average age of 57.4 years. Patients were randomly divided into two groups using the random number table method: control group (n=60), patients who received cerebral artery stents alone; treatment group (n=60), patients who received stents combined with microcatheter cerebral artery injection of tirofiban. This study was conducted in accordance with the declaration of Helsinki and approved by the local Ethics Committee.

### Interventional therapy and medication methods

Patients were admitted to the hospital as soon as possible through the green channel (emergency medical service), those with surgical contraindications were excluded, and a signed informed consent was provided at the same time. Furthermore, blood pressure and water electrolyte and acid-base balance were maintained during the preoperative preparation. Emergency head CT scan ruled out bleeding, and head CT did not reveal any obvious low density lesions. In addition, these patients underwent cranial MRI, diffusion weighted imaging (DWI), and MR angiography (MRA) or head CT angiography (CTA) and were confirmed to have cerebral artery occlusion. Immediately after agreement by the patient or authorized family member, DSA and comprehensive endovascular treatment were performed.

Patients were placed in the supine position before the operation, and anesthesia was determined according to the degree of coordination. If the patient could cooperate, merely local anesthesia was given. If the patient was in a poor state of consciousness and could not easily cooperate, general anesthesia was given. The majority of patients adopted the endovascular treatment under local anesthesia using the modified Seldinger technique; the puncture was performed at the right or left femoral artery and a 6F arterial sheath was placed. Using the imaging tube, total cerebral angiography was first performed to understand the collateral compensatory situation, and the lesion site was assessed.

**Figure 1.**
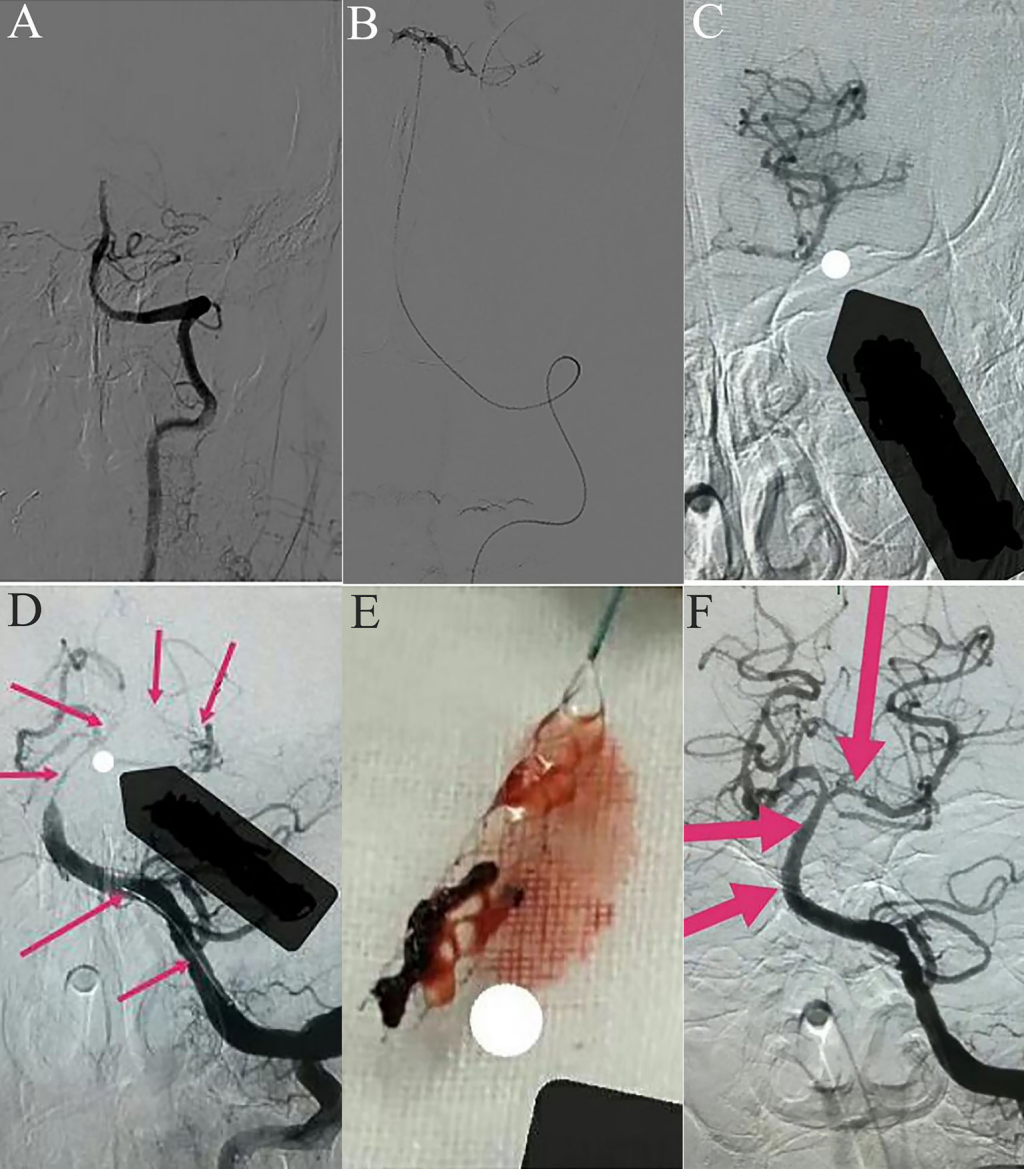
A, Occlusion of the distal end of the preoperative basilar artery. **B**, Microcatheter tip reaches the distal end of the thrombus, and the microcatheter is clearly defined in the vascular cavity. **C**, Microcatheter imaging shows good blood flow in the distal end of the artery. **D**, Stent was placed in the lesion. **E**, Stent removed the thrombus. **F**, Basilar artery and its branches were unobstructed.

If blood vessel or subtotal occlusions were found and the conditions were appropriate, intravascular treatment was attempted (Figure 1A).

Peripheral venous heparinization was performed with a heparin dosage of 60 U/kg, and a 6F guided catheter tip was placed into the responsible artery (vertebral artery or carotid artery) under the guidance of a super-slip guidewire. On the road map, the rebar-18 or rebar-27 (EV3 Inc., USA) microcatheter tip was navigated to reach the distal end of the thrombus. The microcatheter was clearly defined in the vascular cavity (Figure 1B).

The microcatheter was treated with contact antithrombotic therapy with tirofiban. Then, the microcatheter was retracted, the head was buried in the thrombus, and the administration of tirofiban was continued. Next, the microcatheter was withdrawn to the proximal end of the thrombus, and tirofiban was injected at a total amount of 0.2 μg/kg per min. After 15 min, the catheter was reexamined. If the artery remained closed, stenting was performed; if it was open, the administration of 0.1 μg/kg per min of tirofiban was continued to the peripheral vein.

#### Endovascular thrombectomy

The microcatheter was inserted through the occluded artery. The microcatheter trace imaging revealed that distal blood flow was good in the arterial lumen (Figure 1C). According to the diameter of the lesion, the Solitaire AB 4×20 mm or Solitaire AB 6×20 mm stent (EV3 Inc., USA) was placed into the lesion (Figure 1D). Then, the stent and microcatheter were simultaneously removed from the guide tube, the catheter was removed from the body, and the thrombus extracted by the Solitaire stent was examined (Figure 1E). When necessary, the procedure was performed several times and an angiogram was performed to determine if the blood vessel had complete recanalization after the thrombectomy was completed (Figure 1F).

If the angiography revealed that the vessel wall and the main branches were smooth and the flow rate was normal (Figure 1F), the thrombectomy was completed.

After the operation, low molecular weight heparin calcium (0.4 mL/day) was subcutaneously injected, and nimoton (5 mL/h) was given to prevent thrombosis and cerebral vasospasm, or tirofiban was continued at 6 mL/h. Systolic pressure was maintained within 110–140 mmHg. If patients were given recombinant tissue plasminogen activator (rt-pa) for preoperative or intraoperative treatment, 100 mg/day of oral aspirin was given within 24 h after the thrombectomy and 75 mg/day of clopidogrel was given for a month, followed by 100 mg/day of long-term oral aspirin. If the patient had not received rt-pa or arterial thrombolytic treatment, aspirin and clopidogrel were immediately given either orally or by nasal feeding. If the balloon dilation and stent implantation were performed, 100 mg/day of aspirin and 75 mg/day of clopidogrel was given for a total of 3–6 months. For intraoperative cases, 300 mg of aspirin and 300 mg of clopidogrel were given through anus, or intraoperative and postoperative continuous pumping at 6 mL/h for a total of six h were given after 3-6 months, 100 mg/day of aspirin or 75 mg/day of clopidogrel was administered for life.

Postoperative cerebral infarction and thrombolysis in cerebral infarction (TICI), and TICI grades <2b were defined as cerebral hypoperfusion. After interventional therapy, the head MRI+DWI+MRA was reviewed, and some patients received a cephalic CTA examination to analyze the recirculation of cerebral vessels. Major adverse cerebrovascular events in the hospital and at 30 days after discharge, including death, non-fatal re-infarction, target blood vessel blood transport, and reconstruction, were recorded. The NIHSS scores were compared before and 7 days after surgery. After discharge, the follow-up was conducted by telephone inquiry and outpatient consultation. The incidence of hospital bleeding events was recorded. At three months after surgery, the head MRI+MRA was reviewed, and mRS was used to assess the prognosis of patients.

### Statistical analysis

Measurement data are reported as means±SD and analyzed by the *t*-test. Counting data are reported as frequency and percentage, and the chi-squared test was used for comparisons between groups. If there were theoretical frequencies <5, the precise probability method was adopted. If the difference between multiple groups was statistically significant, the comparison between two groups was based on the chi-squared test. A P-value <0.05 was considered statistically significant. SPSS 21.0 (USA) was used for statistical analysis.

## Results

Comparison of clinical baseline data of the two groups of patients is presented in Table 1. The differences in gender, age, clinical features, cerebral artery lesion characteristics, stroke to femoral artery puncture time, intravenous thrombolysis time, and the placement of stents were not statistically significant.

**Table 1.** Comparison of clinical baseline data between the two groups.

Item	Treatment group (n=60)	Control group (n=60)	P value
Age	56.4±13.3	58.1±15.6	0.392
Male	42 (70.0)	45 (75.0)	0.448
Hypertension	36 (60.0)	38 (63.3)	0.783
Diabetes	13 (21.6)	10 (16.7)	0.651
Smoking	24 (40.0)	21 (35.0)	0.470
Family stroke history	9 (15.0)	7 (11.7)	0.388
Medical history of TIA	5 (0.08)	4 (0.07)	0.233
Systolic blood pressure (mmHg)	130±14.6	139±16.4	0.319
Diastolic blood pressure (mmHg)	75±12.2	74±11.9	0.311
LDL-C (mmol/L)	2.4±1.2	2.5±0.9	0.229
Creatinine (μmol/L)	72±14.9	76±15.7	0.338
Blood glucose at admission (mmol/L)	9.4±3.4	8.8±3.6	0.322
Intravenous thrombolysis	17 (28.3)	19 (31.6)	0.864
The baseline NIHSS	14.3±6.4	15.6±7.1	0.388
Atrial fibrillation	16 (26.7)	18 (30.0)	0.163
Coronary heart disease	9 (15.0)	7 (11.7)	0.140
Middle cerebral artery occlusion	29 (48.3)	27 (45.0)	0.191
Internal carotid artery occlusion	5 (8.3)	6 (10.0)	0.266
Vertebral basilar artery occlusion	26 (43.3)	27 (45.0)	0.315
Duration of stroke to femoral artery puncture	4.6±3.1	4.3±2.8	0.330
Number of stents (pieces)	0.26±0.02	0.23±0.01	0.359
Stent diameter (mm)	5.34±0.12	5.21±0.11	0.561
Stent length (mm)	25.24±1.2	24.3±1.4	0.453

Data are reported as mean±SD or number and percentage. TIA: transient ischemic attack; LDL-C: low density lipoprotein cholesterol. Data were compared with the *t*-test or chi-squared test.

Therapeutic effect of interventional therapy is shown in Table 2. The proportion of patients with postoperative TICI 2b/3 was higher in the treatment group compared to the control group (88.3 (53/60) and 66.7% (40/60), P=0.036). Seven days after surgery, the treatment group had lower NIHSS scores than the control group (P=0.048). Furthermore, the 90-day prognosis was better, compared to patients who did not use tirofiban (P=0.024), and these two groups did not have any recurrence of cerebral infarction and blood vessel reconstruction.

**Table 2.** Comparison of therapeutic effect between the two groups.

	Treatment group (n=60)	Control group (n=60)	P value
TICI (2b/3)	53 (88.3)	40 (66.7)	0.036
Postoperative cerebral hemorrhage	7 (11.7)	8 (13.3)	0.452
Symptomatic intracranial hemorrhage	2 (3.3)	1 (1.7)	0.367
Reocclusion	1 (1.7)	5 (8.3)	0.019
Stent thrombosis	0 (0)	1 (7.1)	0.179
90d mRS (0–2)	37 (61.7)	27 (45.0)	0.024
7d NIHSS	4.39±3.24	6.26±4.10	0.048
Mortality	2 (3.3)	3 (5.0)	0.223

Data are reported as mean±SD or number and percentage. TICI: thrombolysis in cerebral infarction score; 90d mRS: modified Rankin scale at 90 days; 7d NIHSS: National Institutes of Health Stroke Scale on day 7 postoperatively. Data were compared with the *t*-test or chi-squared test.

Postoperative cerebral hemorrhage occurred in seven (11.7%) patients in the control group, of which one patient had symptomatic cerebral hemorrhage. In the treatment group, eight (13.3%) patients had post-thrombectomy cerebral hemorrhage, of which two patients had symptomatic cerebral hemorrhage. There was no statistically significant difference in postoperative bleeding (P=0.452) and symptomatic cerebral hemorrhage (P=0.367) between the two groups (Table 2).

During hospitalization and follow-up, three patients died in the control group, two of cerebral hernia at two days after the thrombectomy and one of pulmonary infection at 10 days after the thrombectomy. In the treatment group, two patients died after the thrombectomy, one of cerebral hernia at three days after the thrombectomy and one of pulmonary infection after the second half of the thrombectomy. The mortality between the two groups was not statistically significant (P=0.223). Moreover, stent thrombosis was not statistically different in the two groups (P=0.179). However, after mechanical thrombolysis, the reocclusion of blood vessels of the control group was significantly higher than the treatment group (P=0.019). Eventually, one patient had cerebral vascular interlayer and one patient had hematoma at the puncture site, while there was no complication related to other neurointerventional techniques.

## Discussion

In the treatment of acute cerebral infarction and intravascular interventional therapy or intravenous thrombolytic therapy, even after the infarcted arterial blood flow is restored to a normal level, some patients continue to have brain cell damage, because although the blood vessel is recanalized, there may be ineffective recanalization causing the effective perfusion of brain cells to be limited. The result is that the prognosis is not ideal after the successful recirculation of intracranial occlusion, and the neurological symptoms are progressive.

The main mechanisms of cerebral infarction include the microcirculation embolization of brain tissues, microcirculation ischemia reperfusion injury, and microvascular structure destruction. In recent years, a number of studies have focused on how to reduce and prevent the microcirculation embolism and blocking of reperfusion injury pathways, such as the oxygen free radical shed, neutrophil aggregation blocking capillaries, cell and interstitial edema, microvascular spasm, endothelial dysfunction, and other aspects of brain protection.

Mechanical removal of the thrombus cannot directly improve microcirculation. Due to the presence of microthrombi, the combination of antiplatelet drugs can improve microcirculation, as platelets play a key role in the process of the early formation of blood clots. Therefore, platelet membrane glycoprotein 2b/3a, which is a receptor antagonist, can theoretically alleviate microthrombus formation and the subsequent damage induced in the microcirculation, improving the level of brain tissue reperfusion. Cerebral angiogram revealed that the culprit lesions were high thrombus-loaded lesions, leading to a high incidence of in-stent thrombosis. The active application of platelet membrane glycoprotein 2/1b I, which is a receptor antagonist, can reduce the possibility of blood vessel occlusion, improving the effectiveness of brain tissue reperfusion.

Tirofiban is a selective platelet glycoprotein 2b/3a antagonist that can inhibit platelet aggregation, thereby preventing the atherosclerosis cerebral infarction that results from the *in situ* thrombosis after occlusion. In addition, the safety of tirofiban for acute cerebral infarction has been verified (9).

Previous studies have found that patients with acute cerebral infarction have a high probability (16–18%) of reocclusion in the treatment of mechanical thrombolysis (10). Therefore, it is of great clinical significance to find a treatment method that can prevent the reocclusion of blood vessels after mechanical thrombolysis.

The lesion vessels of acute cerebral infarction tend to have high thrombus load and more atherosclerotic plaque fragments, and the drug therapy has certain limitations in these aspects. Cerebrovascular stents should be mainly applied in the culprit or vessels with larger diameters, completely isolating the responsible thrombotic intracranial artery in patients. This can directly pull out suspensions in the stent thrombosis and thrombogenic substances (the lesion location release of inflammatory mediators and vascular active substance). With the mechanical treatment of acute cerebral infarction, the 90-day prognosis of 90 patients significantly improved (11). However, some cases were unsuccessful due to the vessels, resulting in poor prognosis. Previous studies have reported that the recirculation rate of the responsible blood vessel after intravascular therapy is 66–76%, while a 90-day good prognosis was 32.6–43.7% (10). In the present study, the vascular recirculation rate of patients in the treatment group was 88.3% and the functional rate was 61.7%.

It was hypothesized that in the treatment of endovascular thrombectomy, most of the reoccluded vessels were related to the formation of *in situ* thrombosis. Hence, in the mechanical process, blood vessel occlusion would occur again within the arteries. Therefore, a low dose of tirofiban should be given to prevent blood vessel blockage. This was supported by the present results. Kim et al. (12) reported that the intra-arterial treatment with tirofiban could reduce the infarct area, improve reperfusion, and reduce the occurrence of reocclusion.

In patients with acute cerebral infarction who received treatment, a high incidence of vascular occlusion occurred. The application of small doses of the drug in the intracranial arteries can prevent reocclusion and improve the prognosis of patients. The present study was conducted within a certain time window. In addition to the vein thrombolysis drug and according to the intraoperative cerebrovascular disease condition, endovascular mechanical thrombolysis or thrombectomy, vascular balloon angioplasty, cerebrovascular stent implantation, and the combined use of these methods was selected (12,13 [Bibr B14] [Bibr B15]).

Endovascular thrombectomy can quickly restore blood flow and improve clinical outcomes. Specifically, the earlier the artery is opened for early recovery of the cerebral blood flow, the greater the chance for patients to recover. The safety of Solitaire AB support extraction remains controversial. Hence, large-scale randomized controlled clinical trials are needed to validate the use of Solitaire AB stents. Furthermore, when compared with thrombectomy alone, it can significantly improve the prognosis of patients (13–16). The group of 120 patients who underwent endovascular thrombectomy obtained good results after stent implantation, confirming that the method can benefit patients with acute ischemic stroke.

Intracerebral hemorrhage is one of the major complications of acute ischemic stroke thrombolysis or intravascular therapy. Intravascular mechanical thrombectomy increases the risk of additional complications, such as vascular perforation, cerebrovascular dissection, and the fracture of thrombectomy devices. The reason for postoperative hemorrhage may include reperfusion thrombolysis drug use and the combination of antiplatelet and anticoagulant therapy (17). In this group of patients, eight patients had hemorrhage after the treatment with tirofiban, and the bleeding rate was approximately 13.3%, which was not significant when compared with the control group. Because of our Center's strict indications for thrombectomy and arterial thrombolysis, and strict postoperative management, the complications are within controllable range.

A stroke patient with a disease course exceeding the time window, a brain CT revealing low density changes or an MRI with a clear infarct, and high preoperative blood pressure (systolic blood pressure >180 mmHg, diastolic blood pressure >100 mmHg), if treated with thrombolysis or thrombectomy, can easily develop cerebral hemorrhage (18). In contrast to drug thrombolysis, intravascular thrombectomy increases the risk of additional complications. The present study suggests that endovascular treatment for acute anterior circulation cerebral infarction within 6–8 hours, and acute posterior circulation cerebral infarction in 12–14 h has a certain feasibility.

In the present study, all selected patients underwent cerebral angiography, which confirmed the complete occlusion of the vessel, and a cerebral vascular stent was placed combined with an intravenous injection of tirofiban. The limitation of the present study was that the number of patients was small. Hence, a study with a larger sample size is needed to further confirm these findings.

## References

[1] De Silva DA, Churilov L, Olivot JM, Christensen S, Lansberg MG, Mlynash M (2011). Greater effect of stroke thromboIysis in presence of arterial obstruction. Ann Neurol.

[2] Mishra NK, Albers GW, Davis SM, Donnan GA, Furlan AJ, Hacke W (2010). Mismatch-based delayed thrombolysis: a meta-analysis. Stroke.

[3] Goyal M, Menon BK, van Zwam WH, Dippel DW, Mitchell PJ, Demchuk AM (2016). Endovascular thrombectomy after large-vessel ischaemic stroke: a meta-analysis of individual patient data from five randomised trials. Lancet.

[4] Qureshi AI, Siddiqui AM, Kim SH, Hanel RA, Xavier AR, Kirmani JF (2004). Reocclusion ofrecanalized arteries during intra-arterial thrombolysis for acute ischemic stroke. AJNR Am J Neuroradiol.

[5] Oh HG, Chung PW, Rhee EJ (2015). Increased risk for intracranial arterial stenosis in the subjects with coronary artery calcification. Stroke.

[6] Kurre W, Aguilar-Perez M, Schmid E, Sperber W, Bazner H, Henkes H (2014). Clinical experience with the pREset stent retriever for the treatment of acute ischemic stroke - a review of 271 consecutive cases. Neuroradiology.

[7] Park JH, Park SK, Jang KS, Jang DK, Han YM (2013). Critical use of balloon angioplasty after recanalization failure with retrievable stent in acute cerebral artery occlusion. J Korean Neurosurg Soc.

[8] Klisch J, Sychra V, Strasilla C, Taschner CA, Reinhard M, Urbach H (2015). Double solitaire mechanical thrombectomy in acute stroke: effective rescue strategy for refractory artery occlusions?. AJNR Am J Neuroradiol.

[9] Siebler M, Hennerici MG, Schneider D, von Reutern GM, Seitz RJ, Röther J (2011). Safety of tirofiban in acute ischemic stroke: the SaTIS trial. Stroke.

[10] Janjua N, Alkawi A, Suri MF, Qureshi AI (2008). Impact of arterial reocclusion and distal fragmentation during thrombolysis among patients with acute ischemic stroke. AJNR Am J Neuroradiol.

[11] Saver JL, Goyal M, Bonafe A, Diener HC, Levy EI, Pereira VM (2015). Stent-retriever thrombectomy after intravenous t-PA vs. t-PA alone in stroke. N Engl J Med.

[12] Kim JW, Jeon P, Kim GM, Bang OY, Byun HS, Kim KH (2012). The Local intraarterial tirofiban after formation of anterograde flow in patients with acute ischemic stroke: preliminary experience and short term follow-up results. Clin Neurol Neurosurg.

[13] Berkhemer OA, Fransen PS, Beumer D, van den Berg LA, Lingsma HF, Yoo AJ (2015). A randomized trial of intraarterial treatment for acute ischemic stroke. N Engl J Med.

[B14] Sobolewski P, Kozera G, Kaźmierski R, Michalak S, Szczuzhniak W, Nyka W (2015). Efficacy of cerebral thrombolysis in an extended ‘time window’. J Clinic Pharm Ther.

[B15] Seifert M, Ahlbrecht A, Dohmen C, Spuentrup E, Moeller-Hartmann W (2011). Combined interventional stroke therapy using intracranial stent and local intra-arterial thrombolysis (LIT). Neuroradiology.

[16] Venker C, Stracke P, Berlit P, Diehl RR, Kurre W, Sorgenfrei U (2010). New options in the therapeutic management of a cute ischaemic stroke. Good results with combined i. v. And i. a. Lysis and mechanical thrombectomy [in German]. Fortschr Neurol Psychiatr.

[17] Demchuk AM, Goyal M, Menon BK, Eesa M, Ryckborst KJ, Kamal N (2015). Endovascular treatment for small core and anterior circulation proximal occlusion with emphasis on minimizing CT to recanalization times (ESCAPE) trial: methodology. Int J Stroke.

[18] Saver JL, Jahan R, Levy EI, Jovin TG, Baxter B, Nogueira RG (2012). Solitaire flow restoration device versus the Merci Retriever in patients with acute ischaemic stroke (SWIFT): a randomised, parallel-group, non-inferiority trial. Lancet.

